# Protective Effects of *Myrtus communis* Essential Oil Against Bisphenol A‐Induced Metabolic Dysfunction‐Associated Fatty Liver Disease in Wistar Rats

**DOI:** 10.1002/fsn3.71520

**Published:** 2026-02-08

**Authors:** Mhimdi Mariem, Selmi Slimen, Amira Zammali, Manel Hraoui, Soumaya Wahabi, Dallacqua Stefano, Sebai Hichem

**Affiliations:** ^1^ Laboratory of Functional Physiology and Valorization of Bio‐Resources Higher Institute of Biotechnology of Beja, University of Jendouba Beja Tunisia; ^2^ Department of Pharmaceutical and Pharmacological Sciences University of Padova Padova Italy

**Keywords:** antioxidant status, bisphenol A, MASLD, metabolic syndrome, *Myrtus communis*

## Abstract

Endocrine disruptors such as bisphenol A (BPA) are increasingly associated with metabolic disorders, including metabolic dysfunction‐associated fatty liver disease (MASLD). This preclinical study investigates the effect of BPA on liver function and lipid metabolism in Wistar rats and evaluated the protective potential of *Myrtus communis* essential oil (EOMC) and vitamin E (Vit E). MASLD was induced in Wistar rats by oral administration of BPA (100 mg/kg/day) for 30 days. Rats were co‐treated with EOMC at doses of 50,100, or 200 mg/kg body weight, or Vit E at 100 mg/kg. Liver and kidney function markers, lipid profiles, oxidative stress parameters, and organ histology were assessed. BPA exposure significantly increased plasma liver enzymes (AST: 195,76 ± 4,47 U/L, ALT: 91,05 ± 0,58 U/L) and lipid levels (triglycerides: 1,30 ± 0,13 g/L) compared to controls, and elevated oxidative stress markers, including malondialdehyde (MDA). Co‐treatment with EOMC improved these parameters in a dose‐dependent manner. At 200 mg/kg, EOMC reduced plasma triglycerides 0,62 ± 0,09 g/L, corresponding to an approximate 52% decrease compared to the BPA‐only group, along with an approximately 84% reduction in hepatic MDA levels. Liver histology confirmed marked attenuation of steatosis and hepatocellular damage. Vitamin E showed similar protective effects, though slightly less pronounced. Both EOMC and Vit E demonstrated potential in mitigating against BPA‐induced MASLD. These findings indicate that EOMC may have a protective effect against fatty liver changes in this experimental model, but further studies are needed to confirm its relevance in humans.

## Graphical abstract

Exposure to bisphenol A (BPA) induced hepatic steatosis associated with metabolic dysfunction (MASLD) in Wistar rats, characterized by dyslipidemia, oxidative stress, and hepatic steatosis. Co‐treatment with essential oil of *myrtus communis* (EOMC) improved markers of liver function, lipid profile, and antioxidant status, with histological improvement.

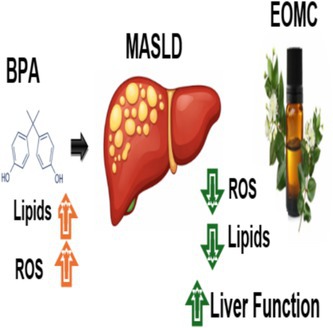

## 1 Introduction

Metabolic dysfunction associated steatotic liver disease (MASLD), recently adopted to replace the term non‐alcoholic fatty liver disease (NAFLD), represents the hepatic manifestation of metabolic dysfunction and is now the most prevalent chronic liver disorder worldwide (Machado 2023, Syed‐Abdul 2023). MASLD affects approximately 40% of adults and is closely associated with obesity, insulin resistance, dyslipidemia, and type 2 diabetes mellitus (Mentsiou Nikolaou et al. 2024). The disease spectrum ranges from simple steatosis to steatohepatitis, fibrosis, and cirrhosis, making MASLD a major nutritional and public health challenge (AbdalHussin et al. 2025).

In the past decade, environmental factors have received a well‐deserved increase in attention due to the parallel rise of both metabolic pathologies and the production or usage of endocrine‐disruptive chemicals (Lința et al. 2024) particularly bisphenol A (BPA), which is a widely used synthetic compound found in polycarbonate plastics and epoxy resins, leading to chronic exposure in humans and animals. BPA exhibits estrogenic activity and has been shown to induce oxidative stress, inflammation, and mitochondrial dysfunction in several organs, especially the liver (Alotaibi et al. 2024). After oral ingestion, BPA is metabolized in the liver into BPA‐G and BPA‐S, which are primarily excreted via bile, urine, and feces, with a half‐life of less than 12 h (Inoue et al. 2001; Mao et al. 2023). However, repeated exposure to moderate or high doses of BPA can overwhelm detoxification pathways, resulting in excessive generation of reactive oxygen species (ROS), lipid peroxidation, and hepatic injury (Hassan et al. 2012; Kobayashi et al. 2020).

Oxidative stress plays a central role in BPA‐induced hepatotoxicity and MASLD progression by enhancing lipid peroxidation, altering antioxidant defenses, and promoting inflammatory responses (Mentsiou Nikolaou et al. 2024; Tang et al. 2024; Yuan et al. 2025). Accordingly, nutritional strategies aimed at reinforcing antioxidant capacity have gained considerable interest. Vitamin E has been extensively investigated as a dietary antioxidant and has demonstrated beneficial effects on liver oxidative status and histological features in MASLD (Gheonea et al. 2025; Vrentzos et al. 2025). However, the search for natural, food‐derived bioactive compounds with hepatoprotective properties remains an important research focus.

Essential oils are increasingly recognized as valuable sources of natural (Bhavaniramya et al. 2019). *Myrtus communis* L., a Mediterranean aromatic plant traditionally used in food preservation and folk medicine, is rich in monoterpenes such as 1,8‐cineole, α‐pinene, linalool, and α‐terpineol (Özkan and Güray 2009; Hennia et al. 2019). These compounds exhibit strong antioxidant and anti‐inflammatory activities (Al‐Harrasi et al. 2022), and it was selected for this study due to their high content of bioactive monoterpenes, making them a promising candidate for hepatoprotection. Despite these promising properties, the protective effect of *Myrtus communis* essential oil against environmentally induced hepatic steatosis, particularly BPA‐related MASLD, has not yet been thoroughly investigated.

This study, therefore, aimed to investigate the potential of EOMC to prevent BPA‐induced MASLD in a rat model, comparing its efficacy to that of Vit E, by assessing metabolic profiles, oxidative stress status, and hepatic histopathology.

## 2 Materials and Methods

### 2.1 Reagents and Chemicals

2,2‐Diphenyl‐1‐picrylhydrazyl (CAS 1898‐66‐4 | Sigma‐Aldrich); 2,2′‐Azino‐bis(3‐ethylbenzothiazoline‐6‐sulfonic acid) (CAS 30931‐67‐0 | Sigma‐Aldrich); Gentamicin sulfate salt (CAS 1405‐41‐0 | Sigma‐Aldrich); Bisphenol A (CAS 80‐05‐7 | Sigma‐Aldrich); Butylated hydroxytoluene (CAS 128‐37‐0 | Sigma‐Aldrich); Trichloroacetic acid (CAS 76‐03‐9 | Sigma‐Aldrich); 2‐thio‐barbituric acid (CAS 84030‐12‐6 | Sigma‐Aldrich); 5,5′‐dithiobis(2‐nitrobenzoic acid) (CAS 69‐78‐3 | Sigma‐Aldrich); Vitamin E was obtained in the form of commercially available capsules from a pharmacy.

### 2.2 Essential Oil Extraction and Analysis

Myrtle leaves were harvested in March in the Hammam Bourguiba region (in the north‐west of Tunisia) and identified by the botanist Chokri Hafsi.

The essential oil was extracted by hydrodistillation using a Clevenger‐type apparatus for 3 h. The distillate was dried over anhydrous sodium sulfate and stored at 4°C until use.

The essential oil yield was calculated as (Mohamadi et al. 2021):

$$\text{Yield of essential oil}=\frac{\text{Weight of the essential oil}}{\text{Weight of the plant used}}\times 100$$

To ensure reproducibility, the extraction was performed in three independent replicates.

#### 2.2.1 GC–MS Analysis

The extracted essential oil of myrtle leaves was analyzed using gas chromatography coupled with mass spectrometry using Trace GC ULTRA/Polaris Q (GC‐MS, Thermo Electron) by the method of Abidi et al., with slight modifications (Abidi et al. 2018).

### 2.3 In Vitro Antioxidant and Antimicrobial Tests

#### 2.3.1 Antioxidant Assays (DPPH and ABTS)

Antioxidant capacity of EOMC was assessed using DPPH and ABTS assays in triplicate, following (Abramovič et al. 2018; Wahabi et al. 2023), with slight modifications.

#### 2.3.2 Antimicrobial Activity

The antibacterial activity of essential oil from *Myrtus Communis* was assessed against Gram‐positive bacteria *Staphylococcus aureus* ATCC 29213, *Enterococcus faecalis* ATCC29212, *Bacillus cereus* (clinical isolate) and *Listeria monocytogenes* and Gram‐negative bacteria ( *Escherichia coli* ATCC 25983, *Salmonella enteritidis* ATCC 13076, and *Pseudomonas aeruginosa* ATCC 27853), using two methods: the disk diffusion method and liquid microdilution method (Dakhli et al. 2025). Gentamicin (10 μg/disc) served as a positive control.

### 2.4 Animal Study Design

All animal procedures were approved by the Biomedical Ethics Committee (CEBM, Pasteur Institute of Tunis, approval no. JORT472001, 15/04/2024) and complied with NIH and ARRIVE guidelines. Healthy male Wistar rats (170–180 g) were housed under standard pet shop conditions (22°C ± 0.5°C, and 12 h/12 h light/dark cycle), with access to food (standard pellet diet‐ Badr Utique‐TN) and water ad libitum. After 2 weeks of acclimatization, rats were randomly assigned to 8 groups (*n* = 6). The sample size (*n* = 6 per group) was determined based on commonly used group sizes in similar preclinical studies of hepatoprotection to ensure adequate power for detecting significant biochemical and histological differences. The animals received oral treatment for 4 weeks. To evaluate the *preventive* potential of EOMC, rats received EOMC or vitamin E for 7 days before and concurrently with BPA administration for the subsequent 30 days. In the following Table 1:

**TABLE 1:** Experimental groups and treatments.

Groups	Treatments
G1	Vehicle control: 0.4 mL/kg/day of corn oil
G2	BPA (100 mg/kg)
G3	EOMC (100 mg/kg)
G4	EOMC (50 mg/kg) + BPA
G5	EOMC (100 mg/kg) + BPA
G6	EOMC (200 mg/kg) + BPA
G7	Vitamin E (100 mg/kg) + BPA
G8	Vitamin E (100 mg/kg)

Vitamin E‐stripped corn oil was prepared by treating commercial corn oil with activated alumina (aluminum oxide) to remove tocopherols, following the method described by Rokosik et al. (2019).

Bisphenol A (100 mg/kg) was selected based on previous studies demonstrating its ability to reliably induce hepatic steatosis and oxidative stress in reflecting a toxicologically relevant exposure in rats (Gurmeet et al. 2014; Kamel and Ahmed 2018).

Note: From the 7th day of treatment, bisphenol A is introduced into groups 4, 5, 6, and 7.

### 2.5 Tissue Collection and Biochemical Analysis

After 4 weeks, rats were fasted overnight and euthanized. Blood, liver, and kidneys were collected for biochemical and histological analyses. Additionally, white adipose tissues, including epididymal (EAT) and subcutaneous (SAT) adipose tissues, were meticulously gathered and weighed.

Plasma biomarkers such as liver enzymes, lipids, and biomarkers of kidney function were measured using certified Biomaghreb kits and a SELECTRA PRO XL analyzer.

Atherogenic index (AI), Coronary risk index (CRI), and the cholesterol‐VLDL were calculated using the following formulas (Kazemi et al. 2018):

- AI = TC‐HDL‐C/HDL
- CRI = TC/HDL‐C
- Chol‐VLDL (Borschovetska and Marchenko 2019) = Chol‐VLDL = TG / 5 × 2,29

### 2.6 Oxidative Stress and Antioxidant Enzyme Assays

Liver and kidney homogenates were prepared for the assessment of MDA, SOD, CAT, GPx, and thiol groups using standard protocols (Aebi 1974; Flohé and Günzler 1984; Kakkar et al. 1984; Draper and Hadley 1990; Hu 1994).

### 2.7 Histopathology

Liver and kidney tissues were fixed, paraffin‐embedded, sectioned (5 μm), and stained with hematoxylin–eosin following (Hajji et al. 2020).

### 2.8 Statistical Analysis

All results were presented as mean ± SD. Statistical analysis was performed using one‐way analysis of variance (ANOVA) with GraphPad Prism statistical software, Version 9.0.2 (GraphPad Software Inc., La Jolla, CA, USA), to compare between groups. When significant differences were found, post hoc comparisons were performed using Tukey's HSD to correct for multiple testing. *P*‐values less than 0.05 were considered statistically significant.

## 3 Results

### 3.1 Composition of EOMC

In this study, the yield of essential oil was 1% in *Myrtus Communis* leaves. The composition of EOMC with the retention times of the compounds are presented in Table 2.

**TABLE 2:** Chemical composition of the essential oil of *Myrtus communis* L. leaves as determined by GC–MS analysis.

Peak	Compounds	Relative content (%)	RT (min)
1	Alpha. ‐Thujene	0.618	6.009
2	Alpha. ‐Pinene	59.749	6.197
3	Beta. ‐Pinene	0.410	7.111
4	Butanoic acid, 2‐methyl‐, 2‐methylpropyl ester	0.260	7.674
5	l‐Phellandrene	0.450	7.739
6	Delta.3‐Carene	0.768	7.879
7	p‐Cymene	2.297	8.214
8	dl‐Limonene	7.020	8.337
9	1,8‐Cineole	18.651	8.393
10	gamma‐Terpinene	0.808	9.030
11	Alpha‐Terpinolene	1.164	9.746
12	Linalool	1.666	10.001
13	Butanoic acid, 2‐methyl‐, 2‐methylbutyl ester	0.435	10.093
14	Alpha. Terpineol	1.241	12.203
15	Linalyl acetate	0.624	13.676
16	α‐Terpinenyl acetate	0.447	15.815
17	Geranyl acetate	1.911	16.515
18	Methyleugenol	0.804	16.996
19	Caryophyllene	0.676	17.413

Abbreviation: RT, Retention time (min).

**FIGURE 1:**
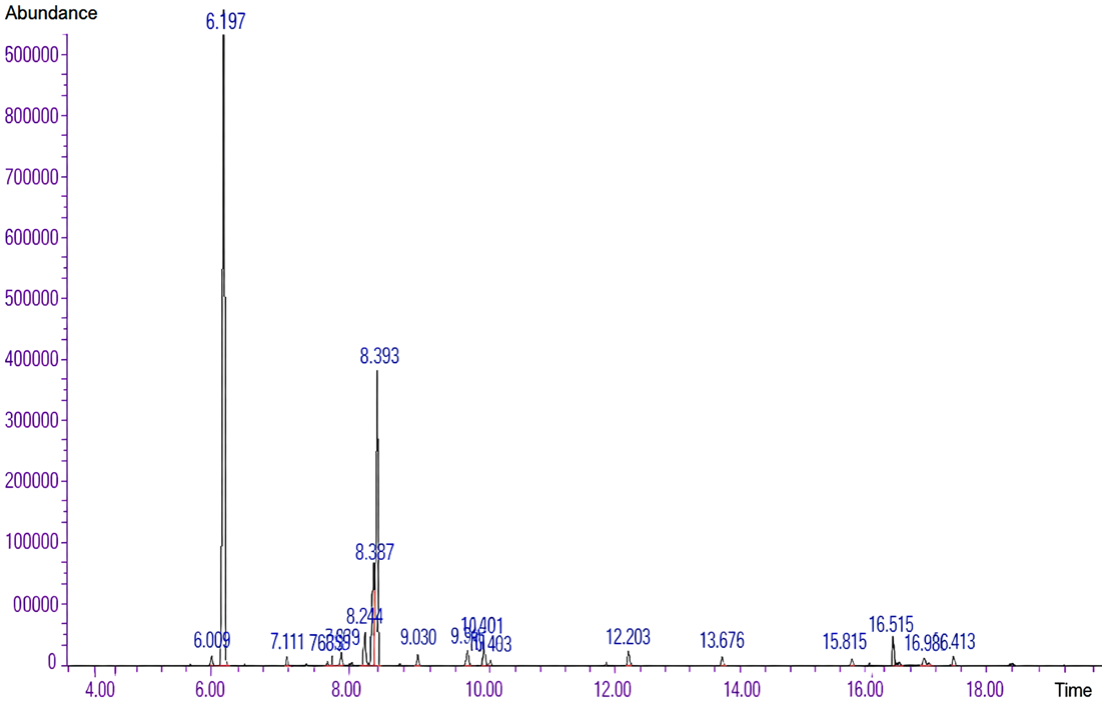
GC–MS chromatogram of the essential oil extracted from the leaves of *Myrtus communis* L., showing the retention times of the identified volatile compounds.

Based on the findings from GC–MS chromatographic analysis (Figure 1), the most abundant constituents present in this oil were alpha‐pinene (59.749%), 1.8‐cineole (18.651%), and d‐Limonene (7.020%).

### 3.2 In Vitro Antioxidant Activity of EOMC

**FIGURE 2:**
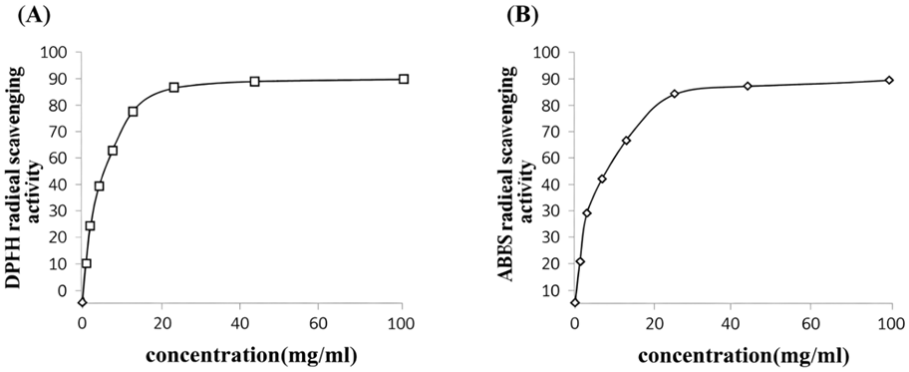
Dose response of the antioxidant capacity of EOMC against the 2,2‐diphenyl‐1 picrylhydrazyl radical (DPPH) (A) and the 2,2‐azino‐bis (3‐ethylbenzothiazoline‐6‐sulfonic acid) radical (ABTS) (B).

EOMC exhibited a significant in vitro antioxidant capacity, as demonstrated by the different assays employed, such as DPPH (Figure 2A) and ABTs (Figure 2B). The oil showed a dose‐dependent free radical scavenging activity, confirming its ability to counteract oxidative stress in vitro.

### 3.3 Antimicrobial Activity of EOMC

The antimicrobial activity of EOMC varied according to the assay method used.

#### 3.3.1 Disk Diffusion Assay

Using the disk diffusion method, EOMC exhibited inhibitory activity against *Bacillus cereus* , with an inhibition zone diameter of 17.66 mm, and against *Escherichia coli* ATCC, with an inhibition zone diameter of 12 mm. No inhibition zones were observed for the other tested bacterial strains (Table 3).

**TABLE 3:** Antibacterial activity of *Myrtus communis* L. essential oil (EOMC) evaluated by the disk diffusion method against Gram‐positive and Gram‐negative bacterial strains. Inhibition zones are expressed as mean ± SD (mm).

Bacterial Strains	Inhibition diameter (mm)	
	EOMC (20 μL/disc)	Gentamicin (10 IU/disc)
Gram (+)		
*Staphylococcus aureus ATCC*	(−)	18
*Enterococcus feacalis ATCC*	(−)	36
*Bacillus cereus*	17.66 ± 2.56	22
*listeria monocytogenes*	(−)	
Gram (−)		
*Escherichia coli ATCC*	12 ± 0	30
*Pseudomonas aeruginosa ATCC*	(−)	18
*Salmonella enteritidis ATCC*	(−)	20

*Note:* Number of bacterial strains = 3.

#### 3.3.2 Broth Microdilution Assay

In contrast, the liquid microdilution method showed that EOMC exerted bacteriostatic activity against all tested Gram‐positive and Gram‐negative bacterial strains (Table 4).

**TABLE 4:** Antibacterial activity of *Myrtus communis* L. essential oil (EOMC) determined by the broth microdilution method against Gram‐positive and Gram‐negative bacterial strains.

Bacterial strains	EOMC		
	CMI (mg/ml)	CMB (mg/ml)	CMB/CMI
Gram (+)			
*Staphylococcus aureus ATCC*	25	200	8
*Enterococcus feacalis ATCC*	6.25	200	32
*Bacillus cereus*	12.5	200	16
*listeria monocytogenes*	1.562	200	128.04
Gram (−)			
*Escherichia coli ATCC*	3.125	200	64
*Pseudomonas aeruginosa ATCC*	12.5	200	10
*Salmonella enteritidis ATCC*	25	200	8

*Note:* Number of bacterial strains = 3.

Abbreviations: MIC, Minimum inhibitory concentration; MBC, minimum bactericidal concentration.

The difference in activity between the disk diffusion and broth microdilution assays may be attributable to the limited diffusion of the hydrophobic essential oil components in the agar matrix, a limitation overcome in the liquid broth medium.

### 3.4 Effects of EOMC and Vit E on Liver and Renal Function Plasma

**FIGURE 3:**
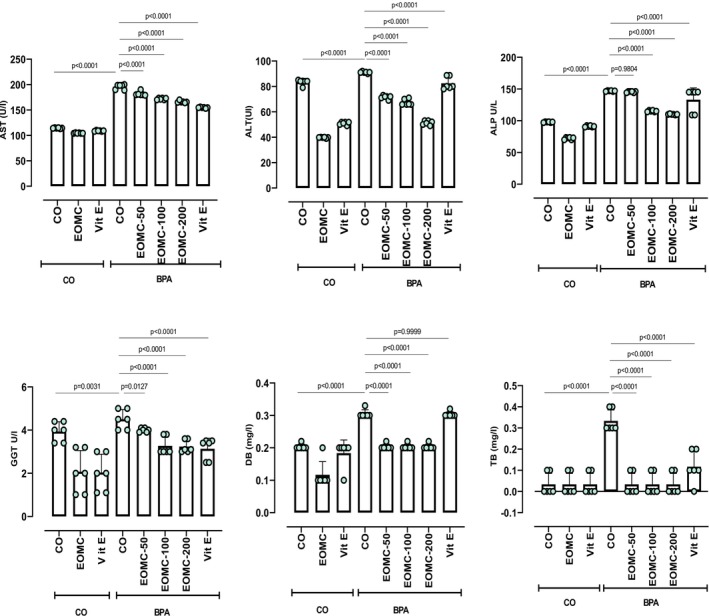
Effects of EOMC and Vit E on plasma liver and renal function parameters in BPA‐exposed rats (*n* = 6 per group). CO: Corn oil negative groups, EOMC: Essential oil of *myrtus comminus*, Vit E: Vitamin E, BPA: Bisphenol A, EOMC‐BPA: Essential oil of *myrtus comminus* at (50,100, and 200 mg/kg) with bisphenol A, Vit E‐BPA: Vitamin E with bisphenol A; AST: Aspartate aminotransferase; ALT: Alanine aminotransferase; ALP: Alkaline phosphatase; GGT: Gamma‐glutamyl transferase; DB: Direct bilirubin; TB: Total bilirubin. Values are the mean ± SD. Significance: *p* values of the compared conditions are indicated.

BPA exposure severely impaired liver function, as evidenced by significant increases in plasma AST, ALT, ALP, DB, and GGT (Figure 3). Co‐treatment with EOMC, particularly at 100 and 200 mg/kg, effectively reversed these changes. However, TB remained unchanged between the groups.

**FIGURE 4:**
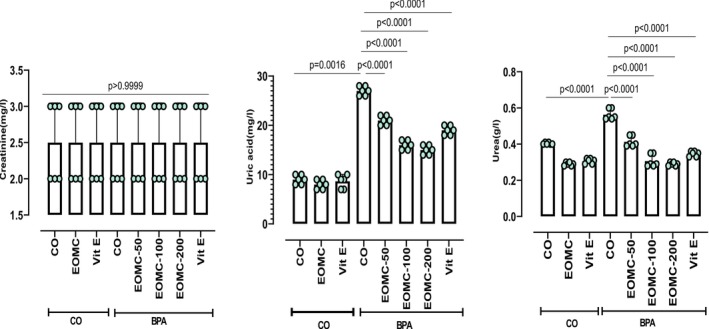
Effect of EOMC and Vit E on levels of creatinine, uric acid, and urea in plasma in rats exposed to BPA. Number of rats: *n* = 6/group. CO: Corn oil negative groups, *EOMC*: Essential oil of *myrtus comminus*, Vit E: Vitamin E, BPA: Bisphenol A, EOMC‐BPA: Essential oil of *myrtus comminus* at (50,100, and 200 mg/kg) with bisphenol A, Vit E‐BPA: Vitamin E with bisphenol A. Values are the mean ± SD. Significance: *p* values of the compared conditions are indicated.

BPA exposure also profoundly affected plasma markers of renal function, as shown by significant increases in plasma uric acid and urea (Figure 4). As expected, co‐treatment with EOMC at different concentrations 50,100 and 200 mg/kg, ameliorated these changes. while creatinine levels remained unaffected.

### 3.5 Effect of EOMC and Vit E on Mineral Levels in Plasma

**FIGURE 5:**
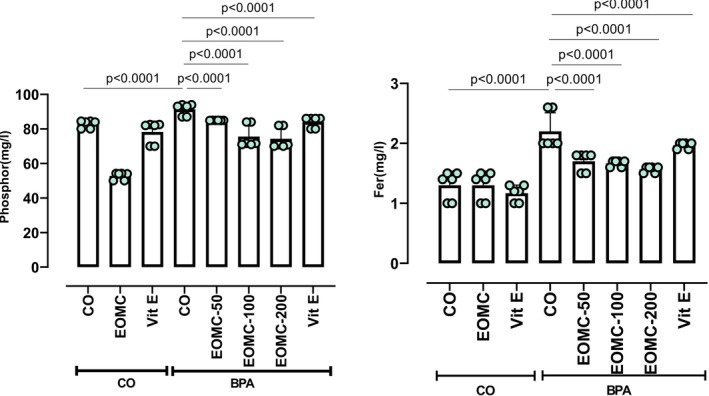
Effect of EOMC and Vit E on mineral levels in plasma in rats exposed to BPA. Number of rats: *n* = 6/group. CO: Corn oil negative groups, *EOMC*: Essential oil of *myrtus comminus*, Vit E: Vitamin E, BPA: Bisphenol A, EOMC‐BPA: Essential oil of *myrtus comminus* at (50,100, and 200 mg/kg) with bisphenol A, Vit E‐BPA: Vitamin E with bisphenol A. Values are the mean ± SD. Significance: *p* values of the compared conditions are indicated.

BPA exposure significantly altered plasma mineral homeostasis, as evidenced by a marked decrease in plasma phosphorus levels and a significant increase in plasma iron concentrations in BPA‐treated rats (Figure 5). Treatment with EOMC at doses of 50,100, and 200 mg/kg, as well as vitamin E, effectively attenuated these alterations.

### 3.6 Effect of EOMC and Vit E on Glucose in Plasma

**FIGURE 6:**
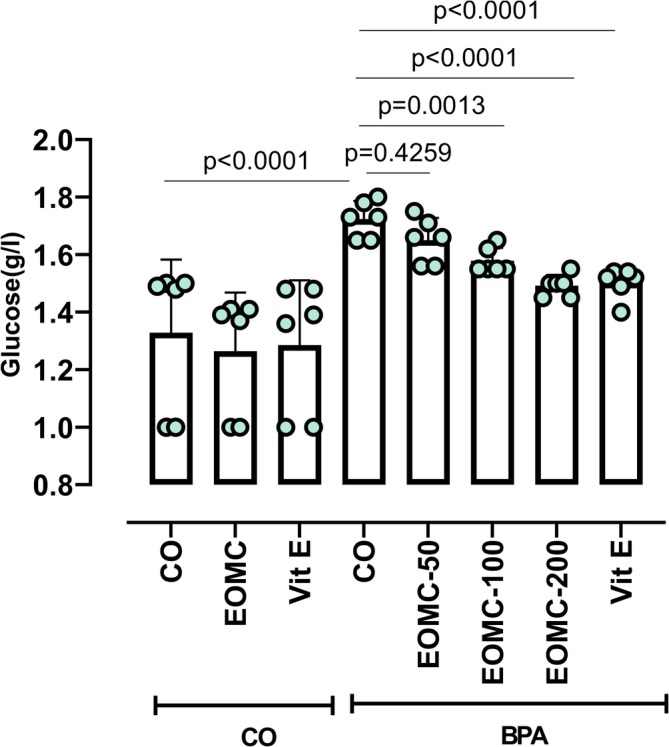
Effect of EOMC and Vit E on plasma glucose in rats exposed to BPA. Number of rats: *n* = 6/group. CO: Corn oil negative groups, EOMC: Essential oil of *myrtus comminus*, Vit E: Vitamin E, BPA: Bisphenol A, EOMC‐BPA: Essential oil of *myrtus comminus* at (50,100, and 200 mg/kg) with bisphenol A, Vit E‐BPA: Vitamin E with bisphenol A. Values are the mean ± SD. Significance: *p* values of the compared conditions are indicated.

BPA exposure stringently exhibited significantly higher plasma glucose concentrations (Figure 6) compared with the control group. EOMC at different concentrations 50,100, and 200 mg/kg effectively modulated this alteration.

### 3.7 Effect of EOMC and Vit E on Lipid Profile

**FIGURE 7:**
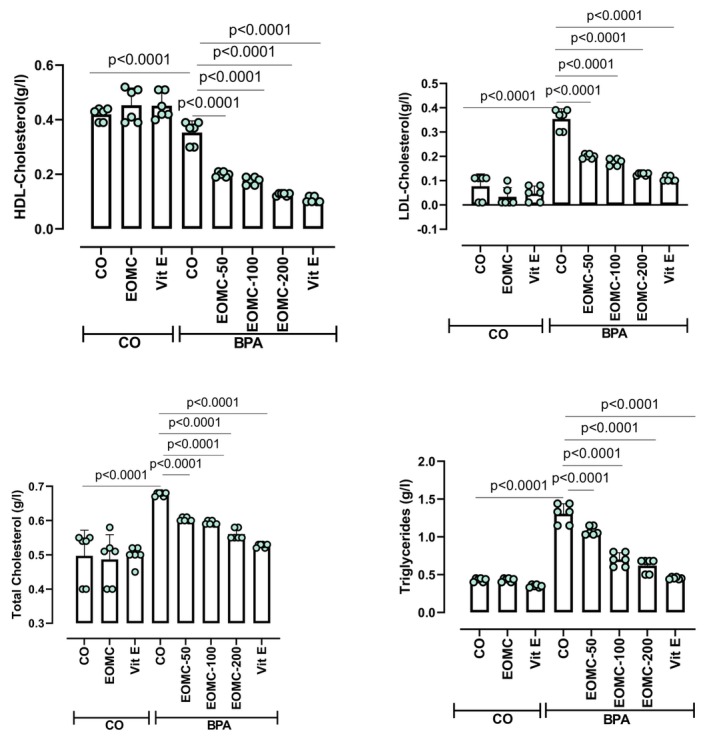
Effect of EOMC and Vit E on serum lipid profile (HDL‐C, LDL‐C, total cholesterol, and triglycerides) in rats exposed to BPA. Number of rats: *n* = 6/group. CO: Corn oil negative groups, EOMC: Essential oil of *myrtus comminus*, Vit E: Vitamin E, BPA: Bisphenol A, EOMC‐BPA: Essential oil of *myrtus comminus* at (50,100, and 200 mg/kg) with bisphenol A, Vit E‐BPA: Vitamin E with bisphenol A, HDL: High‐density lipoprotein; LDL: Low‐density lipoprotein. Values are the mean ± SD. Significance: *p* values of the compared conditions are indicated.

BPA exposure markedly disrupted the plasma lipid profile (triglycerides, total cholesterol, LDL‐C, and HDL‐C) (Figure 7). EOMC at different concentrations 50,100, and 200 mg/kg effectively attenuated these changes.

**FIGURE 8:**
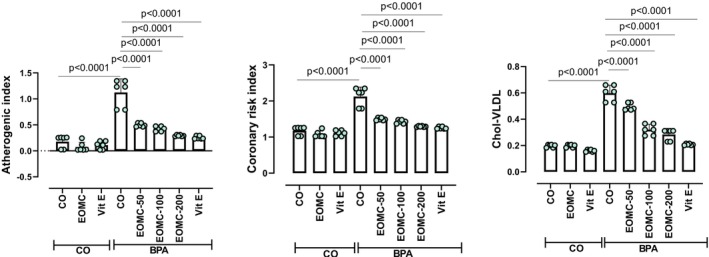
Effects of EOMC and vit E on the atherogenic index (AI), Coronary risk index (CRI), and very low‐density lipoprotein cholesterol (VLDL‐C) in rats exposed to BPA. Number of rats: *n* = 6/group. CO: Corn oil negative groups, EOMC: Essential oil of *myrtus comminus*, Vit E: Vitamin E, BPA: Bisphenol A, EOMC‐BPA: Essential oil of *myrtus comminus* at (50,100, and 200 mg/kg) with bisphenol A, Vit E‐BPA: Vitamin E with bisphenol A, AI: Atherogenic index; CRI: Coronary risk index; VLDL‐C: Very‐low‐density lipoprotein cholesterol. Values are the mean ± SD. Significance: *p* values of the compared conditions are indicated.

BPA exposure led to a significant increase in the atherogenic index (AI), cardiac risk index (CRI), and very low‐density lipoprotein cholesterol (VLDL‐C) levels (Figure 8). As expected, EOMC at different doses and Vit E effectively decreased AI, CRI, and VLDL‐C levels in BPA‐intoxicated Wistar rats.

### 3.8 Effect of EOMC and Vit E on Weight Organs and Adipose Tissue

**FIGURE 9:**
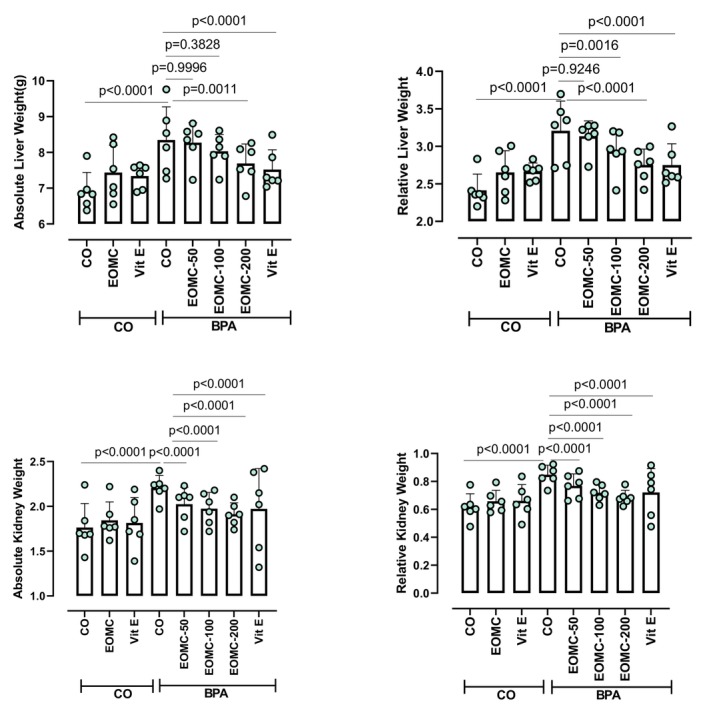
Effect of EOMC and Vit E on absolute and relative weight of organs in rats exposed to BPA. Number of rats: *n* = 6/group. CO: Corn oil negative groups, EOMC: Essential oil of *myrtus comminus*, Vit E: Vitamin E, BPA: Bisphenol A, EOMC‐BPA: Essential oil of *myrtus comminus* at (50,100, and 200 mg/kg) with bisphenol A, Vit E‐BPA: Vitamin E with bisphenol A. Values are the mean ± SD. Significance: *p* values of the compared conditions are indicated.

**FIGURE 10:**
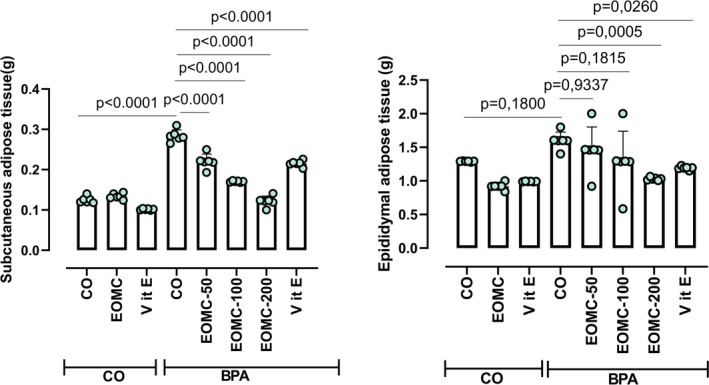
Effect of EOMC and Vit E on subcutaneous and epididymal adipose tissue in rats exposed to BPA. Number of rats: *n* = 6/group. CO: Corn oil negative groups, EOMC: Essential oil of *myrtus comminus*, Vit E: Vitamin E, BPA: Bisphenol A, EOMC‐BPA: Essential oil of *myrtus comminus* at (50,100, and 200 mg/kg) with bisphenol A, Vit E‐BPA: Vitamin E with bisphenol A. Values are the mean ± SD. Significance: *p* values of the compared conditions are indicated.

At the end of the experiment, on the day of sacrifice, the weights of the liver (Figure 9), epididymal, and subcutaneous fat tissues (Figure 10) were measured. Rats exposed to BPA exhibited significantly higher liver and fat tissue weights compared with the control group. As anticipated, administration of EOMC at different concentrations 50,100, and 200 mg/kg, and Vit E significantly reduced liver, epididymal, and subcutaneous fat weights compared with the BPA‐treated group (Figures 9 and 10).

### 3.9 Effect of EOMC and Vit E on Liver and Kidneys on Lipid Peroxidation

**FIGURE 11:**
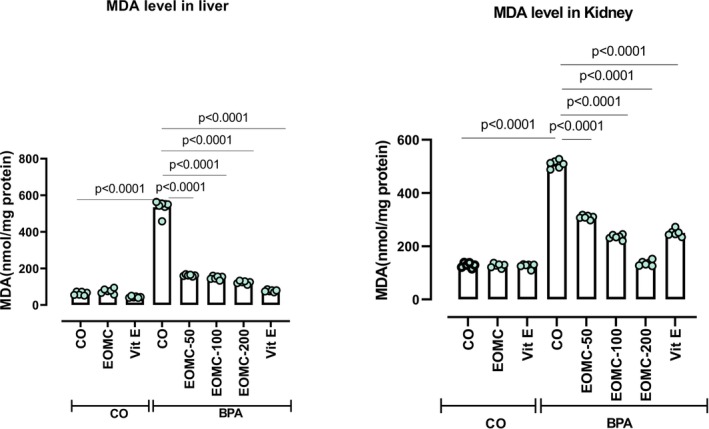
Effect of EOMC and Vit E on malondialdehyde (MDA) in liver and kidney in rats exposed to BPA. Number of rats: *n* = 6/group. CO: Corn oil negative groups, EOMC: Essential oil of *myrtus comminus*, Vit E: Vitamin E, BPA: Bisphenol A, EOMC‐BPA: Essential oil of *myrtus comminus* at (50,100, and 200 mg/kg) with bisphenol A, Vit E‐BPA: Vitamin E with bisphenol A. Values are the mean ± SD. Significance: *p* values of the compared conditions are indicated.

Rats treated with BPA exhibited significantly higher malondialdehyde (MDA) levels in the liver and kidneys (Figure 11). Consistent with expectations, treatment with EOMC Specifically at 100 and 200 mg/kg, and vit E significantly decreased MDA levels with greater reductions observed at higher doses compared with the BPA‐treated group.

### 3.10 Effect of EOMC and Vit E on the Activity of Antioxidant Enzymes

**FIGURE 12:**
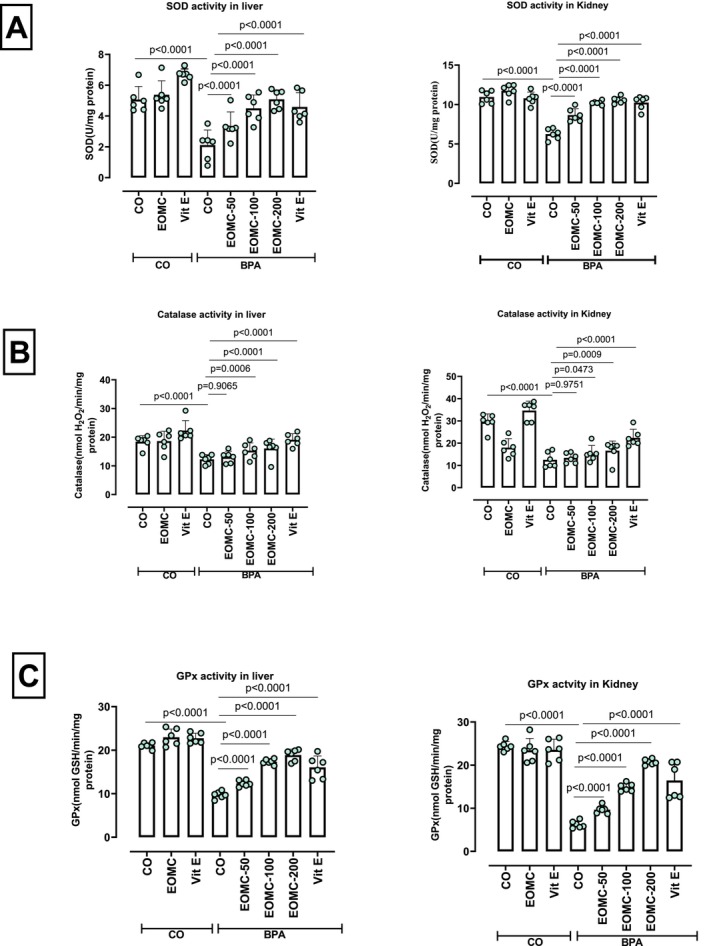
Effect of EOMC and Vit E on the activity of antioxidant enzymes (SOD, CAT, and GPx) in rats exposed to BPA. Number of rats: *n* = 6/group. CO: Corn oil negative groups, EOMC: Essential oil of *myrtus comminus*, Vit E: Vitamin E, BPA: Bisphenol A, EOMC‐BPA: Essential oil of *myrtus comminus* at (50,100, and 200 mg/kg) with bisphenol A, Vit E‐BPA: Vitamin E with bisphenol A. Values are the mean ± SD. Significance: *p* values of the compared conditions are indicated.

As shown in Figure 12, BPA‐treated rats exhibited a significant reduction in the activity of superoxide dismutase (SOD) (Figure 12A), catalase (CAT) (Figure 12B), and glutathione peroxidase (GPx) (Figure 12C). Co‐treatment with EOMC was more pronounced at 100 and 200 mg/kg, and Vit E effectively reversed these changes.

### 3.11 Effect of EOMC and Vit Eon the Variation in the Level of Thiol Groups

**FIGURE 13:**
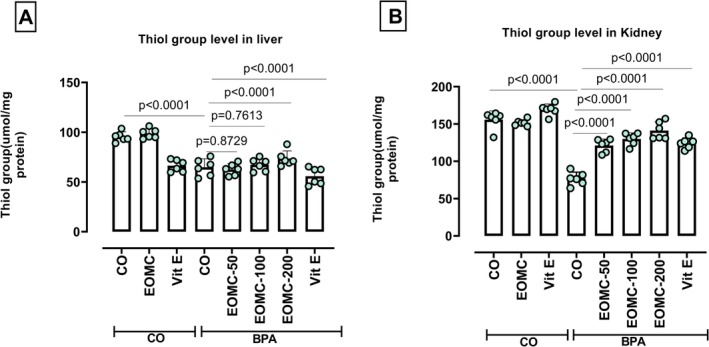
Effect of EOMC and Vit Eon the variation in the level of thiol groups in rats exposed to BPA. Number of rats: *n* = 6/group. CO: Corn oil negative groups, EOMC: Essential oil of *myrtus comminus*, Vit E: Vitamin E, BPA: Bisphenol A, EOMC‐BPA: Essential oil of *myrtus comminus* at (50,100, and 200 mg/kg) with bisphenol A, Vit E‐BPA: Vitamin E with bisphenol A. Values are the mean ± SD. Significance: *p* values of the compared conditions are indicated.

Rats treated with BPA (100 mg/kg) exhibited a marked decrease in thiol group levels in the liver (Figure 13A) and kidneys (Figure 13B). Co‐treatment with EOMC at all tested doses (50,100, and 200 mg/kg) as well as with vit E significantly restored thiol group levels, with greater reductions observed at higher doses compared with the BPA‐treated group.

### 3.12 Histopathological Changes in Liver and Kidney Tissues

**FIGURE 14:**
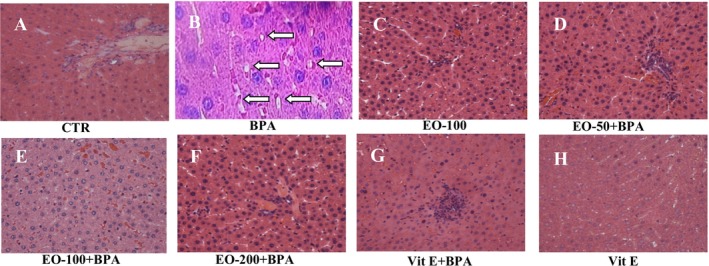
Histopathological changes in liver tissues following bisphenol a exposure and protective effects of myrtle essential oil and vitamin E. Representative liver tissue sections stained with hematoxylin and eosin (H&E), magnification 400×. (A) Control group showing normal liver histology with intact hepatocytes and sinusoids. (B) BPA‐treated group displayed marked hepatocyte swelling, sinusoidal congestion (white arrow), necrosis, and inflammatory cell infiltration (star), indicative of severe liver damage. (C, D, E) Groups treated with BPA and Myrtle essential oil at 50,100, and 200 mg/kg, respectively, demonstrated a dose‐dependent improvement: Reduced necrosis and inflammation (stars), with near‐complete restoration of normal architecture at 200 mg/kg. (F) BPA + Vitamin E‐treated group showed significant protection with reduced inflammation and preserved hepatocyte structure. (G) Positive control (Vitamin E only), exhibiting normal liver morphology. (H) Negative control (Myrtle essential oil only) also displays intact histological features.

**FIGURE 15:**
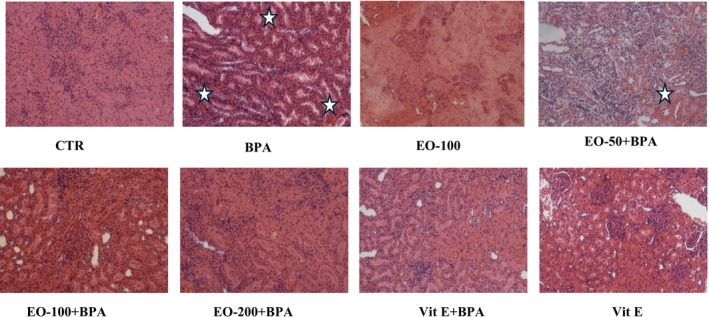
Histopathological changes in kidney tissues following bisphenol a exposure and protective effects of myrtle essential oil and vitamin E. Representative kidney tissue sections stained with hematoxylin and eosin (H&E), magnification 200×. (A) Control group showing normal kidney histology with intact glomeruli and tubules. (B) BPA‐treated group displayed severe tubular necrosis, glomerular alterations, and interstitial inflammation (stars), indicative of significant nephrotoxicity. (C, D, E) Groups treated with BPA and Myrtle essential oil at 50,100, and 200 mg/kg, respectively, demonstrated a dose‐dependent protective effect: Reduced tubular damage and inflammation, with near‐complete restoration of normal kidney morphology at 200 mg/kg. (F) BPA + Vitamin E‐treated group showed marked recovery with reduced necrosis and improved glomerular and tubular structure. (G) Positive control (Vitamin E only), exhibiting normal renal morphology. (H) Negative control (Myrtle essential oil only) also displays intact renal architecture.

Histopathological analysis revealed significant liver (Figure 14) and kidney (Figure 15) damage in the BPA‐exposed group, characterized by hepatocyte swelling, sinusoidal congestion, necrosis, inflammatory infiltration, tubular necrosis, and glomerular alterations, indicative of severe oxidative stress. Co‐treatment with EOMC was associated with a progressive reduction in the severity of these histological alterations, with more preserved tissue architecture observed at higher doses. Similarly, rats co‐treated with vitamin E displayed fewer histopathological alterations in both liver and kidney tissues compared with the BPA‐treated group.

## 4 Discussion

During this study, we demonstrated that EOMC exerts a protective effect against hepatic steatosis associated with BPA‐MASLD in rats. Exposure to BPA resulted in biochemical, oxidative, and histological alterations consistent with hepatic steatosis and liver dysfunction, while co‐administration of myrtle oil significantly attenuated these disturbances. These results suggest that EOMC can limit the development of MASLD‐like features in this experimental model. Importantly, this protection appears to be multifactorial, involving improvements in lipid metabolism, attenuation of oxidative stress, and preservation of hepatic histoarchitecture.

One of the central features of BPA has been associated with marked disruption of plasma lipid homeostasis (Fang et al. 2022), reflecting a dyslipidemic profile characteristic of MASLD development (Mosca et al. 2024; Romeo et al. 2025). The attenuation of these parameters following preventive treatment with EOMC and Vit E suggests an improvement in lipid metabolism and a potential reduction of BPA‐induced lipotoxicity. This protective effect was more pronounced at high doses of EOMC, demonstrating a dose‐dependent relationship. These results are consistent with previous studies showing that BPA promotes lipid accumulation and contributes to hepatic steatosis (Lin et al. 2017; Figueiredo et al. 2020). Furthermore, similar lipid‐lowering and hepatoprotective effects of EOMC have been described in experimental models of metabolic and oxidative liver injury, suggesting a protective role against xenobiotic‐induced dyslipidemia (Odeh et al. 2022).

Oxidative stress is a well‐established contributor to MASLD progression, playing a central role in hepatocellular injury and disease progression (Mignini et al. 2024; Jiang et al. 2025; Miller et al. 2025). BPA exposure is known to promote reactive oxygen species generation and disrupt antioxidant defenses in hepatic tissue (Bindhumol et al. 2003; Wang et al. 2019). In the current study, BPA‐induced oxidative imbalance was evidenced by increased lipid peroxidation and altered antioxidant defenses, whereas EOMC administration was associated with partial restoration of redox balance. This antioxidant effect may be attributed to the bioactive constituents of EOMC, which have been reported to possess free radical scavenging and antioxidant properties in both in vitro and in vivo models (Mimica‐Dukić et al. 2010; Giampieri et al. 2020). These effects could be linked to the chemical composition of EOMC, particularly its chemotype, such as alpha pinene, which may modulate oxidation processes in experimental models (Rahimi et al. 2023, 2025). Also, alpha pinene is an inhibitor of cytochrome P450 (Zehetner et al. 2019), as several studies suggest that the generation of free radicals by CYP2E1 contributes to MASLD (Hardwick and Garcia 2024), especially given that CYP2E1 becomes more toxic in the presence of BPA (Zaulet et al. 2017). Although such mechanisms were not directly assessed in this study, the observed attenuation of oxidative damage supports the hypothesis that reduction of oxidative stress may contribute to the hepatoprotective effects of EOMC in this experimental model.

Histological analysis showed that EOMC administration attenuated BPA‐induced structural alterations in the liver, suggesting that the observed protection is not limited to biochemical or antioxidant changes but may also involve preservation of liver architecture. These observations are like previous studies on experimental models of MASLD that have shown that the combination of lipid improvement and reduced oxidative stress contributes to the histological protection of the liver (Eweda et al. 2020; Salau et al. 2025).

It should be acknowledged that the current study has some limitations. Indeed, no formal sample size calculation was performed before the experiment; the number of animals used was determined based on previous similar studies and practical considerations. Also, this work was conducted in a single experimental setting with male Wistar rats, which may limit the generalizability of the results to other models, including females or other species. Moreover, the exclusive use of males introduces a sex bias, and the selected BPA dose may not fully reflect environmental exposures. Additionally, multiple comparisons increase the risk of type I errors. Future studies should consider including both sexes, testing different animal models, and isolating the main active compounds of *Myrtus communis* essential oil to better understand its protective effects.

## 5 Conclusion

In summary, EOMC, like Vit E, may effectively regulate fatty liver and related disorders by improving plasma lipid levels, reducing oxidative stress, and supporting hepatic structural integrity. This study underscores the detrimental effects of BPA on liver and kidney tissues, leading to oxidative stress and significant structural damage, as confirmed by histopathological analyses. Co‐treatment with EOMC has a dose‐dependent protective effect, restoring normal histology at 200 mg/kg, likely due to its strong antioxidative properties. Vit E also has beneficial effects in reducing oxidative damage. Although these results suggest a potential protective role of EOMC against BPA‐induced toxicity, further studies are warranted, including the use of chronic low‐dose BPA exposures that better reflect real environmental conditions and the isolation of the main active compounds (α‐pinene and 1,8‐cineole) to elucidate the underlying molecular mechanisms.

## Author Contributions

Mariem Mhimdi, Slimen Selmi, Amira Zammali, Manel Hraoui: Investigation: Roles/Writing – original draft; Conceptualization; Supervision; Methodology, Slimen Selmi, Stefano Dallacquaand Hichem Sebai: Project administration; Software; Resources, Writing – review and editing.

## Funding

This research did not receive any specific grant from funding agencies in the public, commercial, or non‐profit sectors. The study was supported by the Higher Institute of Biotechnology of Beja, University of Jendouba, Tunisia, through the provision of laboratory facilities and materials.

## Ethics Statement

All animal procedures were approved by the biomedical ethics committee of the Pasteur Institute of Tunis (approval no. JORT472001, April 15, 2024) and were conducted in accordance with the National Institutes of Health (NIH) guidelines and the ARRIVE guidelines for animal research.

## Conflicts of Interest

The authors declare no conflicts of interest.

## Data Availability

The data that support the findings of this study are available upon request from the corresponding author. The data are not publicly available due to privacy or ethical restrictions.

## References

[fsn371520-bib-0001] AbdalHussin, E. A. , Z. Abd Hamid , M. H. Md Idris , M. H. Omar , and I. S. Taib . 2025. “Bisphenol F and Steatotic Liver Disease: Resolving the PXR Paradox Through Stress Pathway Mechanisms.” Biomedicine 14, no. 1: 30. 10.3390/biomedicines14010030 PMC12839251 41595567

[fsn371520-bib-0002] Abidi, A. , E. Sebai , M. Dhibi , et al. 2018. “Chemical Analyses and Anthelmintic Effects of *Artemisia campestris* Essential Oil.” Veterinary Parasitology 263: 59–65. 30389026 10.1016/j.vetpar.2018.10.003

[fsn371520-bib-0003] Abramovič, H. , B. Grobin , N. S. Ulrih , et al. 2018. “Relevance and Standardization of in Vitro Antioxidant Assays: ABTS, DPPH, and Folin–Ciocalteu.” Journal of Chemistry 2018, no. 1: 4608405.

[fsn371520-bib-0004] Aebi, H. 1974. “Catalase.” In Methods of Enzymatic Analysis, 673–684. Elsevier.

[fsn371520-bib-0005] Al‐Harrasi, A. , S. Bhatia , P. B. Sharma , et al. 2022. “Anti‐Inflammatory, Antioxidant, and Immunomodulatory Effects of EOs.” In Role of Essential Oils in the Management of COVID‐19, 239–255. CRC Press.

[fsn371520-bib-0006] Alotaibi, K. S. , M. Elobeid , P. Virk , et al. 2024. “Plausible Effect of Hesperetin and Nano‐Hesperetin Against Bisphenol‐A Induced Hepatoxicity in a Rat Model.” Arabian Journal of Chemistry 17, no. 2: 105563.

[fsn371520-bib-0007] Bhavaniramya, S. , A. Vishnupriya , M. Saleh Al‐Aboody , et al. 2019. “Role of Essential Oils in Food Safety: Antimicrobial and Antioxidant Applications.” Grain & Oil Science and Technology 2, no. 2: 49–55.

[fsn371520-bib-0008] Bindhumol, V. , K. C. Chitra , and P. P. Mathur . 2003. “Bisphenol A Induces Reactive Oxygen Species Generation in the Liver of Male Rats.” Toxicology 188, no. 2–3: 117–124. 12767684 10.1016/s0300-483x(03)00056-8

[fsn371520-bib-0009] Borschovetska, V. , and М. Marchenko . 2019. “Lipid Profile of Blood Serum in Mice Under Conditions of Bisphenol A Administration and Vitamin A Different Suplementation.” Науковий вісник Чернівецького університету. Біологія (Біологічні системи) 11, no. 2: 115–121.

[fsn371520-bib-0010] Dakhli, N. , A. López‐Jiménez , C. Cárdenas , et al. 2025. “ *Urtica dioica* Aqueous Leaf Extract: Chemical Composition and in Vitro Evaluation of Biological Activities.” International Journal of Molecular Sciences 26, no. 3: 1220. 39940988 10.3390/ijms26031220 PMC11818644

[fsn371520-bib-0011] Draper, H. H. , and M. Hadley . 1990. “Malondialdehyde Determination as Index of Lipid Peroxidation.” Methods in Enzymology 186: 421–431. 2233309 10.1016/0076-6879(90)86135-i

[fsn371520-bib-0012] Eweda, S. M. , A. S. A. Newairy , H. M. Abdou , and A. S. Gaber . 2020. “Bisphenol A‐Induced Oxidative Damage in the Hepatic and Cardiac Tissues of Rats: The Modulatory Role of Sesame Lignans.” Experimental and Therapeutic Medicine 19, no. 1: 33–44. 31853270 10.3892/etm.2019.8193 PMC6909485

[fsn371520-bib-0013] Fang, R. , S. Yang , X. Gu , C. Li , N. Bi , and H. L. Wang . 2022. “Early‐Life Exposure to Bisphenol A Induces Dysregulation of Lipid Homeostasis by the Upregulation of SCD1 in Male Mice.” Environmental Pollution 304: 119201. 35341816 10.1016/j.envpol.2022.119201

[fsn371520-bib-0014] Figueiredo, L. S. , K. M. Oliveira , I. N. Freitas , et al. 2020. “Bisphenol‐A Exposure Worsens Hepatic Steatosis in Ovariectomized Mice Fed on a High‐Fat Diet: Role of Endoplasmic Reticulum Stress and Fibrogenic Pathways.” Life Sciences 256: 118012. 32593710 10.1016/j.lfs.2020.118012

[fsn371520-bib-0015] Flohé, L. , and W. A. Günzler . 1984. “Assays of Glutathione Peroxidase.” Methods in Enzymology 105: 114–120. 6727659 10.1016/s0076-6879(84)05015-1

[fsn371520-bib-0016] Gheonea, D.‐I. , C. Tocia , V.‐M. Sacerdoțianu , et al. 2025. “Therapeutic Efficacy of Silymarin, Vitamin E, and Essential Phospholipid Combination Therapy on Hepatic Steatosis, Fibrosis, and Metabolic Parameters in MASLD Patients: A Prospective Clinical Study.” International Journal of Molecular Sciences 26, no. 12: 5427. 40564891 10.3390/ijms26125427 PMC12192674

[fsn371520-bib-0017] Giampieri, F. , D. Cianciosi , T. Y. Forbes‐Hernández , et al. 2020. “Myrtle (*Myrtus communis* L.) Berries, Seeds, Leaves, and Essential Oils: New Undiscovered Sources of Natural Compounds With Promising Health Benefits.” Food Frontiers 1, no. 3: 276–295.

[fsn371520-bib-0018] Gurmeet, K. , I. Rosnah , M. K. Normadiah , S. das , and A. M. Mustafa . 2014. “Detrimental Effects of Bisphenol A on Development and Functions of the Male Reproductive System in Experimental Rats.” EXCLI Journal 13: 151–160. 26417249 PMC4464354

[fsn371520-bib-0019] Hajji, N. , D. Wannes , M. A. Jabri , et al. 2020. “Purgative/Laxative Actions of Globularia Alypum Aqueous Extract on Gastrointestinal‐Physiological Function and Against Loperamide‐Induced Constipation Coupled to Oxidative Stress and Inflammation in Rats.” Neurogastroenterology & Motility 32, no. 8: e13858. 32337785 10.1111/nmo.13858

[fsn371520-bib-0020] Hardwick, J. P. , and V. Garcia . 2024. “The Synergistic and Opposing Roles of ω‐Fatty Acid Hydroxylase (CYP4A11) and ω‐1 Fatty Acid Hydroxylase (CYP2E1) in Chronic Liver Disease.” Genome Biology & Molecular Genetics 1, no. 1: 15–26. 40452921 10.17352/gbmg.000003 PMC12124931

[fsn371520-bib-0021] Hassan, Z. K. , M. A. Elobeid , P. Virk , et al. 2012. “Bisphenol A Induces Hepatotoxicity Through Oxidative Stress in Rat Model.” Oxidative Medicine and Cellular Longevity 2012, no. 1: 194829. 22888396 10.1155/2012/194829 PMC3409570

[fsn371520-bib-0022] Hennia, A. , S. Nemmiche , S. Dandlen , and M. G. Miguel . 2019. “ *Myrtus communis* Essential Oils: Insecticidal, Antioxidant and Antimicrobial Activities: A Review.” Journal of Essential Oil Research 31, no. 6: 487–545.

[fsn371520-bib-0023] Hu, M.‐L. 1994. “Measurement of Protein Thiol Groups and Glutathione in Plasma.” Methods in Enzymology 233: 380–385. 8015473 10.1016/s0076-6879(94)33044-1

[fsn371520-bib-0024] Inoue, H. , H. Yokota , T. Makino , A. Yuasa , and S. Kato . 2001. “Bisphenol A Glucuronide, a Major Metabolite in Rat Bile After Liver Perfusion.” Drug Metabolism and Disposition 29, no. 8: 1084–1087. 11454725

[fsn371520-bib-0025] Jiang, Z. , L. Chen , and X. Dou . 2025. “Glutathionylation and Metabolic Dysfunction‐Associated Steatotic Liver Disease.” Biochimie 234: 10–19. 40147581 10.1016/j.biochi.2025.03.006

[fsn371520-bib-0026] Kakkar, P. , B. das , and P. N. Viswanathan . 1984. “A Modified Spectrophotometric Assay of Superoxide Dismutase.” Indian Journal of Biochemistry & Biophysics 21, no. 2: 130–132. 6490072

[fsn371520-bib-0027] Kamel, A. H. , and E. T. Ahmed . 2018. “The Adverse Effects of Bisphenol A on Male Albino Rats.” Journal of Basic and Applied Zoology 79, no. 1: 6.

[fsn371520-bib-0028] Kazemi, T. , M. Hajihosseini , M. Moossavi , M. Hemmati , and M. Ziaee . 2018. “Cardiovascular Risk Factors and Atherogenic Indices in an Iranian Population: Birjand East of Iran.” Clinical Medicine Insights: Cardiology 12: 1179546818759286. 29497341 10.1177/1179546818759286 PMC5824902

[fsn371520-bib-0029] Kobayashi, K. , Y. Liu , H. Ichikawa , S. Takemura , and Y. Minamiyama . 2020. “Effects of Bisphenol A on Oxidative Stress in the Rat Brain.” Antioxidants 9, no. 3: 240. 32187996 10.3390/antiox9030240 PMC7139612

[fsn371520-bib-0030] Lin, Y. , D. Ding , Q. Huang , et al. 2017. “Downregulation of miR‐192 Causes Hepatic Steatosis and Lipid Accumulation by Inducing SREBF1: Novel Mechanism for Bisphenol A‐Triggered Non‐Alcoholic Fatty Liver Disease.” Biochimica et Biophysica Acta (BBA)‐molecular and Cell Biology of Lipids 1862, no. 9: 869–882. 28483554 10.1016/j.bbalip.2017.05.001

[fsn371520-bib-0031] Lința, A. V. , F. Cioffi , R. Senese , et al. 2024. “Liver and Pancreatic Toxicity of Endocrine‐Disruptive Chemicals: Focus on Mitochondrial Dysfunction and Oxidative Stress.” International Journal of Molecular Sciences 25, no. 13: 7420. 39000526 10.3390/ijms25137420 PMC11242905

[fsn371520-bib-0032] Machado, M. V. 2023. “MASLD Treatment—A Shift in the Paradigm Is Imminent.” Frontiers in Medicine 10: 1316284. 38146424 10.3389/fmed.2023.1316284 PMC10749497

[fsn371520-bib-0033] Mao, W. , L. Mao , F. Zhou , et al. 2023. “Influence of Gut Microbiota on Metabolism of Bisphenol A, a Major Component of Polycarbonate Plastics.” Toxics 11, no. 4: 340. 37112567 10.3390/toxics11040340 PMC10144690

[fsn371520-bib-0034] Mentsiou Nikolaou, E. , I. Gigante , L. Giannini , et al. 2024. “The Interplay Between Endocrine‐Disrupting Chemicals and the Epigenome Towards Metabolic Dysfunction‐Associated Steatotic Liver Disease: A Comprehensive Review.” Nutrients 16, no. 8: 1124. 38674815 10.3390/nu16081124 PMC11054068

[fsn371520-bib-0035] Mignini, I. , L. Galasso , G. Piccirilli , et al. 2024. “Interplay of Oxidative Stress, Gut Microbiota, and Nicotine in Metabolic‐Associated Steatotic Liver Disease (MASLD).” Antioxidants 13, no. 12: 1532. 39765860 10.3390/antiox13121532 PMC11727446

[fsn371520-bib-0036] Miller, D. M. , K. McCauley , and K. J. Dunham‐Snary . 2025. “Metabolic Dysfunction‐Associated Steatotic Liver Disease (MASLD): Mechanisms, Clinical Implications and Therapeutic Advances.” Endocrinology, Diabetes & Metabolism 8, no. 6: e70132. 10.1002/edm2.70132 PMC12627968 41255342

[fsn371520-bib-0037] Mimica‐Dukić, N. , D. Bugarin , S. Grbović , et al. 2010. “Essential Oil of *Myrtus communis* L. as a Potential Antioxidant and Antimutagenic Agents.” Molecules 15, no. 4: 2759–2770. 20428077 10.3390/molecules15042759 PMC6257387

[fsn371520-bib-0038] Mohamadi, Y. , T. Lograda , M. Ramdani , et al. 2021. “Chemical Composition and Antimicrobial Activity of *Myrtus communis* Essential Oils From Algeria.” Biodiversitas Journal of Biological Diversity 22, no. 2: 1023905.

[fsn371520-bib-0039] Mosca, A. , M. Manco , M. R. Braghini , et al. 2024. “Environment, Endocrine Disruptors, and Fatty Liver Disease Associated With Metabolic Dysfunction (MASLD).” Metabolites 14, no. 1: 71. 38276306 10.3390/metabo14010071 PMC10819942

[fsn371520-bib-0040] Odeh, D. , N. Oršolić , M. Berendika , et al. 2022. “Antioxidant and Anti‐Atherogenic Activities of Essential Oils From *Myrtus communis* L. and *Laurus nobilis* L. in Rat.” Nutrients 14, no. 7: 1465. 35406078 10.3390/nu14071465 PMC9003404

[fsn371520-bib-0041] Özkan, A. M. G. , and Ç. G. Güray . 2009. “A Mediterranean: *Myrtus communis* L.(Myrtle).” In Plants and Culture: Seeds of the Cultural Heritage of Europe, edited by J. P. Morel and A. M. Mercuri , 159–168. Edipuglia.

[fsn371520-bib-0042] Rahimi, K. , A. Rezaie , M. Hatamnezhad , A. Ziyaei , and M. J. Alimohammadi . 2025. “Alpha‐Pinene Protects Rat Liver Against Acetaminophen‐Induced Oxidative Stress and Apoptosis.” Naunyn‐Schmiedeberg's Archives of Pharmacology 398, no. 10: 14057–14066. 40261348 10.1007/s00210-025-04168-x

[fsn371520-bib-0043] Rahimi, K. , M. Zalaghi , E. G. Shehnizad , G. Salari , F. Baghdezfoli , and A. Ebrahimifar . 2023. “The Effects of Alpha‐Pinene on Inflammatory Responses and Oxidative Stress in the Formalin Test.” Brain Research Bulletin 203: 110774. 37793595 10.1016/j.brainresbull.2023.110774

[fsn371520-bib-0044] Rokosik, E. , K. Dwiecki , M. Rudzińska , A. Siger , and K. Polewski . 2019. “Column Chromatography as a Method for Minor Components Removal From Rapeseed Oil.” Grasas y Aceites 70, no. 3: e316.

[fsn371520-bib-0045] Romeo, M. , M. Dallio , F. Di Nardo , et al. 2025. “Exploring the Classic and Novel Pathogenetic Insights of Plastic Exposure in the Genesis and Progression of Metabolic Dysfunction‐Associated Steatotic Liver Disease (MASLD).” Liver 5, no. 2: 21.

[fsn371520-bib-0046] Salau, E. A. D. H. , D. Diglio , G. R. Guimarães , O. V. Furtado‐Filho , and M. Porawski . 2025. “Bisphenol A Alters the Expression of Genes Involved in Lipogenesis, Inflammation, and Oxidative Stress in the Liver of Adult Zebrafish.” Pharmaceuticals 18, no. 11: 1765. 41305006 10.3390/ph18111765 PMC12655367

[fsn371520-bib-0047] Syed‐Abdul, M. M. 2023. “Lipid Metabolism in Metabolic‐Associated Steatotic Liver Disease (MASLD).” Metabolites 14, no. 1: 12. 38248815 10.3390/metabo14010012 PMC10818604

[fsn371520-bib-0048] Tang, J. , K. Wang , D. Shen , and C. Li . 2024. “Oxidative Stress and Keap1‐Nrf2 Pathway Involvement in Bisphenol a‐Induced Liver Damage in Rats.” Toxics 12, no. 12: 864. 39771079 10.3390/toxics12120864 PMC11678961

[fsn371520-bib-0049] Vrentzos, E. , G. Pavlidis , E. Korakas , et al. 2025. “Nutraceutical Strategies for Metabolic Dysfunction‐Associated Steatotic Liver Disease (MASLD): A Path to Liver Health.” Nutrients 17, no. 10: 1657. 40431398 10.3390/nu17101657 PMC12113997

[fsn371520-bib-0050] Wahabi, S. , K. Rtibi , A. Atouani , and H. Sebai . 2023. “Anti‐Obesity Actions of Two Separated Aqueous Extracts From Arbutus ( *Arbutus unedo* ) and Hawthorn ( *Crataegus monogyna* ) Fruits Against High‐Fat Diet in Rats via Potent Antioxidant Target.” Dose‐Response 21, no. 2: 15593258231179904. 37275393 10.1177/15593258231179904 PMC10236257

[fsn371520-bib-0051] Wang, K. , Z. Zhao , and W. Ji . 2019. “Bisphenol A Induces Apoptosis, Oxidative Stress and Inflammatory Response in Colon and Liver of Mice in a Mitochondria‐Dependent Manner.” Biomedicine & Pharmacotherapy 117: 109182. 31387175 10.1016/j.biopha.2019.109182

[fsn371520-bib-0052] Yuan, J. , H. Xu , J. Gan , and H. Zhao . 2025. “Linking Bisphenol a Exposure to MASLD: Insights From Network Toxicology and Machine Learning Based on NHANES 2005–2012 Data.” Toxicology Research 14, no. 6: tfaf165. 41306725 10.1093/toxres/tfaf165 PMC12646254

[fsn371520-bib-0053] Zaulet, M. , S. E. M. Kevorkian , S. Dinescu , et al. 2017. “Protective Effects of Silymarin Against Bisphenol A‐Induced Hepatotoxicity in Mouse Liver.” Experimental and Therapeutic Medicine 13, no. 3: 821–828. 28450905 10.3892/etm.2017.4066 PMC5403334

[fsn371520-bib-0054] Zehetner, P. , M. Höferl , and G. Buchbauer . 2019. “Essential Oil Components and Cytochrome P450 Enzymes: A Review.” Flavour and Fragrance Journal 34, no. 4: 223–240.

